# Lessons Learned From Using Focus Groups to Refine Digital Interventions

**DOI:** 10.2196/resprot.4404

**Published:** 2015-07-31

**Authors:** Jillian LS Avis, Trevor van Mierlo, Rachel Fournier, Geoff DC Ball

**Affiliations:** ^1^Department of PediatricsFaculty of Medicine & DentistryUniversity of AlbertaEdmonton, ABCanada; ^2^Evolution Health Systems IncToronto, ONCanada; ^3^Research AssociateHenley Business SchoolUniversity of ReadingOxfordshireUnited Kingdom; ^4^Pediatric Centre for Weight & HealthStollery Children's HospitalEdmonton, ABCanada

**Keywords:** data collection, digital interventions, focus groups, health care, Internet, qualitative research

## Abstract

There is growing interest in applying novel eHealth approaches for the prevention and management of various health conditions, with the ultimate goal of increasing positive patient outcomes and improving the effectiveness and efficiency of health services delivery. Coupled with the use of innovative approaches is the possibility for adverse outcomes, highlighting the need to strategically refine digital practices prior to implementation with patients. One appropriate method for modification purposes includes focus groups. Although it is a well-established method in qualitative research, there is a lack of guidance regarding the use of focus groups for digital intervention refinement. To address this gap, the purpose of our paper is to highlight several lessons our research team has learned in using focus groups to help refine digital interventions prior to use with patients.

## Introduction

### Background

Digital interventions have an important role to play in promoting health and well-being among patients. However, this mode of delivering information and interaction is not without pitfalls [1]; a reality that highlights the importance of developing and refining interventions in a thoughtful, systematic manner prior to implementation [2]. One available method for refining digital interventions is focus groups, an approach used traditionally in the fields of marketing and advertising research to solicit consumer feedback on concepts and products [3]. Focus groups, now a frequently used method in qualitative research, are unique in that they enable the collection and analysis of three complementary forms of data -individual and group level data, and data generated based on participant interaction [4]. This feature is valuable because the researcher can explore multiple units of analysis to understand the research question. Additionally, focus groups are advantageous as they often allow for the spontaneous discussion of topics (eg, Butler, 1996 [5]) that may otherwise go unvoiced in other methods of data collection, such as individual interviews.

Focus groups have been used to assess individuals’ perceptions of and refinements for changes to the structure, content, and utility of digital interventions. For example, focus groups have been applied to study single, standalone interventions [6,7], educational resources for patients [8,9], and the usability of several comparable tools [10]. Despite these examples, there remains a lack of guidance for using focus groups in the context of digital health, and specifically, digital intervention refinement. To date, most recommendations have emphasized the use of focus groups for nondigital interventions [11] and recruiting participants into focus groups [12]. To address this gap, our purpose was to highlight several lessons that we learned from our collective experience [13-15] in using focus groups to help develop and refine digital interventions.

### Lessons Learned

In a recent study that has been registered with ClinicalTrials.gov (NCT02330588) [13], our research team used focus groups to refine a newly developed online screening, brief intervention, and referral to treatment program designed to enhance parents’ awareness of and motivation to change children’s healthy lifestyle behaviors. The following are practical lessons learned from conducting these focus groups.

### 1. Use a Checklist to Plan, Track, and Report Aspects of the Focus Group

As qualitative research involves the exploration of complex phenomena, explicit and comprehensive reporting can be a challenge. An additional hurdle is clearly articulating the research team’s background, study design, coding process, and key findings, which may be particularly important when researchers acting as focus group moderators are intellectually and potentially financially invested in the digital intervention under study. For transparency and to enhance methodological rigor, a checklist can help to organize and articulate all of the relevant processes and procedures the research team undertook in their research with focus groups. For example, the Consolidated Criteria for Reporting Qualitative Research (COREQ) [16] is a 32-item checklist that can be used to report criteria in three domains: research team and reflexivity (eg, researchers’ credentials, relationship(s) with participants); study design (eg, theoretical framework, participant selection); and analysis and findings (eg, methodology, use of verification strategies).

### 2. Have a Helper

Participants can perceive focus groups for refining digital interventions as opportunities to share their thoughts and opinions about the intervention as well as query the rationale for different intervention elements. However, the focus group moderator has a demanding position to facilitate the flow of discussion and strategically channel participant’s feedback, often within a predetermined time period. Therefore, he/she needs to strike a balance between respectfully allowing participants to “tell their stories” and contribute meaningfully while adhering to their interview guide that is typically designed to solicit feedback on a range of issues related to the intervention. With this in mind, the inclusion of an assistant or collaborator in the focus group can help to keep everyone on time and on task, as well as alleviate the moderator of distracting and time-consuming tasks, such as note-taking. For instance, if the discussion is running long or the group tends to get side-tracked by one or two individuals, the assistant or collaborator might say: “Unfortunately we are running short of time; could we follow up with you regarding your thoughts at a later point?” This strategy allows the moderator to maintain their emphasis on the interview questions and process as well as complete the focus group in a timely manner.

### 3. Prepare for Constructive Feedback

In contrast to many traditional focus groups, which are often used to explore and solicit perspectives related to abstract and conceptual phenomena, focus groups for refining digital interventions are more targeted, querying participants’ opinions on a tangible product in which the researchers (often including the focus group facilitator) may have painstakingly developed. It is not unusual for research team members to have an emotional response to criticism when blood, sweat, and tears have been generated through the intervention development phase. It is essential to prepare oneself for unexpected remarks as the moderator’s negative expressions and/or feedback may unduly sway participants from communicating their true thoughts and feelings, which may compromise the credibility and usefulness of the data.

### 4. Tailor Questions to Participants

It is valuable to obtain perspectives from a diverse group of stakeholders when developing a new intervention. For instance, if developers plan to target substance abuse behaviors in adolescents, it makes sense to solicit feedback from adolescents themselves (the target audience), but also other relevant stakeholders (eg, health care professionals, parents, teachers) who may have a keen interest in the tool or who may play a role in referring or recommending the intervention to adolescents. Depending on the degree of homogeneity in each focus group, moderating questions and facilitating probes may need to be tailored for language and content. In our experience, we tailored discussion questions to groups of parents and health care professionals who were more interested in practical issues (eg, diversity of information and health services to promote healthy nutrition in families) versus researchers who showed a greater affinity for academic elements (eg, assessing parents’ motivation constructs that can predict behavior change) of the intervention.

### 5. Preserve Context When Capturing Data

Unlike focus groups in which participants are encouraged to discuss intangible concepts (eg, an experience or process), focus groups for refining digital interventions typically query participants’ views on concrete elements (eg, aesthetics, ease, and logic of navigation). Given this difference, capturing the discussion of focus group participants with a digital audio recorder and subsequent transcription may not preserve the context of intervention details to which participants refer (eg, “I like the font and images you used on this page”). To improve the accuracy of data capture in focus groups, Scott et al (2009) [17] proposed real-time data transcription using certified court reporters that include transcribing focus group discussions into text, similar to processes used in court hearings and depositions. We have used this approach and realized several benefits, including (1) the transcription is highly accurate; (2) additional context can be included into transcripts if desired; (3) turnaround is quick (3-4 business days), enabling concurrent data collection and analysis, an important tenet of qualitative research [18] even if several focus groups are planned over a short period of time; and (4) the moderator can focus his/her full attention on facilitating the group discussion without concern for data collection.

### 6. Assess the Current Intervention–Do Not Create a New One

Developing or white-boarding unique concepts for digital interventions can be exciting and it is not atypical for focus group members who are highly-engaged to suggest the addition of digital elements outside the scope of the current intervention (eg, incorporation of avatars, chat rooms, and other social media components). An important task of the moderator is to manage and concentrate participants’ feedback to the task at hand. Particularly when refining an intervention, as much of the design, structure, and functional elements have already been established, it is important to stay focused on more proximal aspects of refinement (eg, likability, feasibility, and utility) of the *current* intervention. It may also be helpful for the moderator to explicitly discuss the objectives of the focus group and the kinds of modifications that are possible before the group discussion begins in order for participants to have clear expectations.

### 7. Leverage the “Digital Expert”

In our experience, focus groups often contain at least one “digital expert”, a member with personal or professional experience in design, information architecture, or computer programming. Depending on the nature of the contributions and how the moderator manages the discussion, the digital expert can exert a positive or negative influence on the group discussion. An attentive moderator can leverage the digital expert to help channel the group discussion on intervention attributes; acknowledging the individual’s experience and expertise as well as utilizing probes to draw out information and insights relevant to the current intervention can engender rapport, respect, and openness throughout the group. Issues that arise beyond the scope of the focus group can be respectfully deferred to a later date, which allows the digital expert to contribute additional information while not detracting from the goal at hand.

## Conclusions

Refining digital interventions using focus groups presents unique challenges and opportunities. Based on our experience to date, we have learned a number of lessons, including (1) transparency of the research process can be facilitated through the use of a checklist to plan, track, and report important aspects of the focus group; (2) some participants may misperceive focus groups as an unimpeded opportunity to discuss the intervention and efforts should be employed to optimize use of time; (3) the moderator may be heavily invested (emotionally and/or financially) in the intervention and should be prepared for critical comments from participants; (4) the refinement process may benefit from a number of different perspectives, so tailoring the discussion questions and probing follow-up questions is advised; (5) special consideration for capturing data is required so that the context of the discussion remains clear and accurate at the data analysis phase; (6) the moderator should specify the purpose, which includes refining the existing intervention rather than developing a new one; and (7) a “digital expert” may be present within the group, so the moderator should plan accordingly to manage individual contributions in order to effectively facilitate the group discussion. These practical lessons may be particularly relevant for clinicians and researchers working to refine new digital interventions. Such a process is likely to increase in frequency as health care delivery evolves to adopt novel interventions designed to optimize patient outcomes and improve health care availability, accessibility, and acceptability.

## Abbreviations

COREQ: Consolidated Criteria for Reporting Qualitative Research
